# The effect of proanthocyanidin and casein phosphopeptide‐amorphous calcium phosphate on the bond strength durability to caries‐affected dentin

**DOI:** 10.1002/cre2.368

**Published:** 2020-12-09

**Authors:** Zahra Jowkar, Maryam Firouzmandi, Sara Tabibi

**Affiliations:** ^1^ Oral and Dental Disease Research Center, Department of Operative Dentistry, School of Dentistry Shiraz University of Medical Sciences Shiraz Iran; ^2^ Under Graduate Student, Department of Operative Dentistry, School of Dentistry Shiraz University of Medical Sciences Shiraz Iran

**Keywords:** caries affected dentin, casein phosphopeptide‐amorphous calcium phosphate, proanthocyanidin, microshear bond strength

## Abstract

**Objectives:**

The aim of this study was to evaluate the effect of proanthocyanidin (PA) and casein phosphopeptide‐amorphous calcium phosphate (CPP‐ACP) paste on the micro‐shear bond strength (μSBS) durability of an etch‐and‐rinse adhesive to caries‐affected dentin (CAD).

**Materials and methods:**

The occlusal surfaces of 80 human molars with occlusal caries were ground to expose flat dentin surfaces with CAD. Then, they were randomly divided into four groups (*n* = 20) according to the CAD pretreatment. The study groups included no pretreatment, pretreatment with CPP‐ACP for 3 min, pretreatment with PA for 1 min, and pretreatment with PA for 1 min followed by CPP‐ACP for 3 min before adhesive application. After restoring the specimens with composite resin, μSBS testing was performed for half of the bonded surfaces in each group after 24 h and the other half was tested after 6 months of water storage and failure mode analysis was performed.

**Results:**

The PA group was associated with a higher μSBS than the control and CPP‐ACP groups after 24 hours (*p* < 0.05). No significant difference was observed regarding the μSBS of the control and the other groups after 24 h (*p* > 0.05). No significant difference was observed regarding the μSBS of the PA and PA + CPP‐ACP groups (*p* > 0.05). The μSBS of the 6‐month specimens was significantly lower than those of the 24‐h specimens for all the groups (*p* < 0.05) except for the PA group which did not exhibit a significant difference between the two times (*p* > 0.05). The most common type of failure was mixed failure.

**Conclusion:**

PA pretreatment could stabilize the CAD‐resin interface and protect degradation over time. The same effect was not observed for CPP‐ACP or PA + CPP‐ACP.

## 1 INTRODUCTION

Nowadays, clinicians try to remove the infected‐dentin and leave behind the caries‐affected dentin (CAD) in the cavity (de Almeida Neves et al., 2011). The cyclic demineralization‐remineralization caries process in CAD results in lower mineral content and more porosity of inter‐tubular dentin compared to normal dentin. Therefore, CAD is expected to be more permeable than normal dentin facilitating the easier and deeper diffusion of acidic conditioners and adhesive monomers (Haj‐Ali et al., 2006). The bond strength of composite resin to CAD can be affected by compositional and morphological changes in CAD compared to normal dentin (Zaki et al., 2016). Some efforts have been made to remineralize the porous and hypomineralized intertubular dentin in CAD. The application of a paste containing casein phosphopeptide‐amorphous calcium phosphate (CPP–ACP) such as MI paste (GC Corporation, Tokyo, Japan) can render a supersaturated state for the bioavailable calcium and phosphate ions in the dental substrate (Poggio et al., 2013). The diffusion of calcium and phosphate ions into the porous lesion and their deposition can result in the remineralization of the partially demineralized crystals (Poggio et al., 2013).

Adhesive‐based restorations undergo degradation over time (Kazemi‐Yazdi et al., 2020). Thus, continuing efforts have focused on stabilizing the collagen matrix to achieve a durable bonding between the CAD and adhesives. Various collagen cross‐linkers have been used to increase the collagen crosslinking in dentin (De‐Paula et al., 2020; M. Firouzmandi, Shafiei, et al., 2019; Srinivasulu et al., 2012). Proanthocyanidin (PA) is one of the polyphenolic compounds which acts as a potent antioxidant (Shafiei et al., 2020). Increased collagen synthesis and the decreased rate of enzymatic degradation of collagen matrices have been attributed to PA (Ku et al., 2007; Macedo et al., 2009). An extraordinary cross‐linking efficacy within very short treatment times has been shown for PA (Y. Liu & Wang, 2013). Also, PA has demonstrated superior inhibitory effects compared with chlorhexidine on endogenous matrix metalloproteinases such as MMP‐2, MMP‐8, and MMP‐9 (D. J. Epasinghe, Yiu, et al., 2013). PA pretreatment could preserve the bond strength of the Adper Single Bond to sound dentin after 12 months (C. S. Castellan, Bedran‐Russo, et al., 2013). Moreover, deep dentin specimens treated with 6.5% PA demonstrated significantly higher bond strength values than those treated with sodium ascorbate (Srinivasulu et al., 2012).

The remineralizing effect of the CPP‐ACP and the anti‐collagenolytic and crosslinking effects of PA would be helpful in preventing the degradation of dentin collagen within the hybrid layer of CAD. Hence, the aim of this in vitro study was to evaluate the effect of CPP‐ACP and PA application on micro‐shear bond strength (μSBS) durability of an etch‐and‐rinse adhesive to CAD.

## 2 MATERIALS AND METHODS

Eighty human extracted molars with occlusal caries extending approximately halfway into the dentin were collected for the study. The research protocol was approved by the Ethics Committee of Shiraz University of Medical Sciences (IR.SUMS.REC.1397.375). All the patients signed an informed consent form. All teeth were stored at 4°C in physiologic saline for no longer than 4 weeks after extraction. After extraction, the teeth were thoroughly washed, scrubbed, and scaled to remove any debris. The teeth were stored in 0.5% chloramine T solution at 4°C for 1 week and then placed in distilled water to be used in less than 1 month. Flat midcoronal dentin surfaces which were perpendicular to the long axis of the teeth were exposed by removing the occlusal enamel and superficial dentin of the crown using a water‐cooled low‐speed cutting machine (Mecatome T201 A, Presi, Grenoble, France). Afterward, a Caries Detector (Kuraray Co, Tokyo, Japan) was used in accordance with the manufacturer's instructions. Wet 600‐grit SiC paper was used to perform further finishing for 20 s under running water. The combined criteria of hardness to the sharp excavator, visual examination, and staining with dye were used to distinguish caries‐affected and normal dentin. Infected dentin removal was repeated for each tooth until the discolored, harder dentin which stained pink remained. This exposed area of dentin was considered as CAD (Paulose & Fawzy, 2017). After sectioning the roots 1 mm below the cementoenamel junction, the samples were mounted in acrylic resin (Acropars; Marlik Co., Tehran, Iran) with the dentin surfaces oriented perpendicular to the bottom of the mold.

The teeth were divided into four equal groups (*n* = 20) according to the surface treatment of CAD. In the control group, Adper Single Bond (SB, 3 M ESPE) adhesive was used according to the manufacturer's instructions after application of the 35% phosphoric acid gel (3 M, ESPE, St. Paul, MN) for 15 s on the surface of the CAD. In the second group, a CPP‐ACP‐containing paste (MI paste; GC Corp, Tokyo, Japan) was applied actively using a brush for 3 min after application of the 35% phosphoric acid gel on the surface of CAD (Doozandeh et al., 2015). After washing the surface of the specimens with water, the adhesive was applied. In group 3, the CAD surfaces were pretreated with PA solution for 1 min and then rinsed with water after application of the 35% phosphoric acid gel and before adhesive application (Paulose & Fawzy, 2017). The PA solution was prepared by dissolving 6.5 g of grape seed extract in the form of powder (Puritans Pride Inc., Oakdale, NY) in 100 ml of distilled water to make a 6.5% proanthocyanidin solution (Srinivasulu et al., 2012). After application of the 35% phosphoric acid gel in group 4, the surface of the CAD was first pretreated by PA for 1 min and rinsed with water. Then, CPP‐ACP paste was applied for 3 min. Afterward, the surfaces of the CAD were rinsed and the adhesive was applied.

**FIGURE 1:**
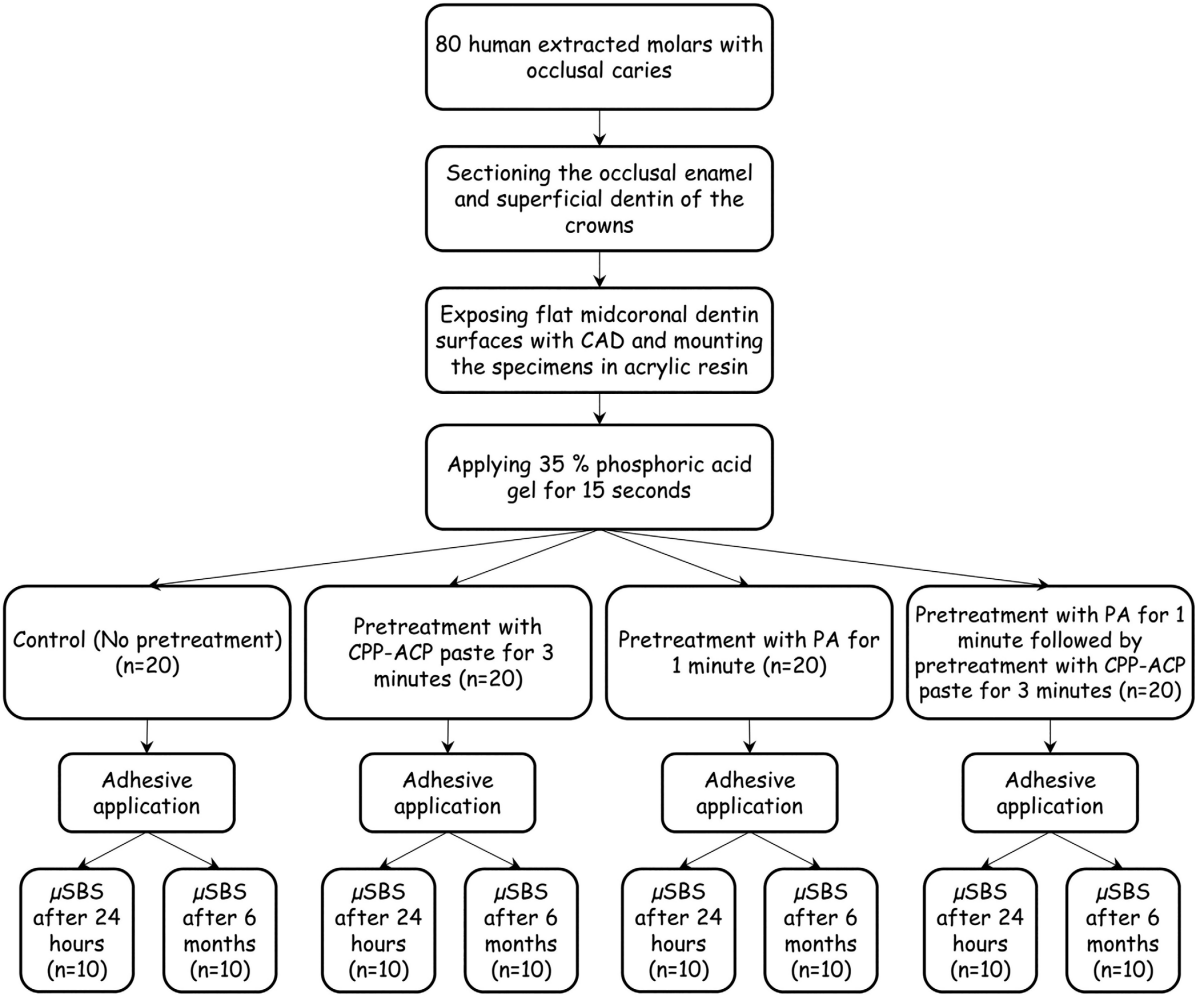
Diagram of the study design. Abbreviations: CAD, Caries affected dentin; CPP‐ACP paste, casein phosphopeptide‐amorphous calcium phosphate paste; PA, proanthocyanidin

A piece of translucent polyvinyl chloride microtube (0.7 mm in internal diameter and approximately 0.5 mm in height) was placed on the CAD surface which had been defined by an adhesive tape with a punched hole over the center of the flattened CAD surface. The microtubes were subsequently filled with Z250 composite (3 M ESPE, St Paul, MN). Light curing was carried out with a light curing unit (VIP Junior, Bisco, Schaumburg, IL) at 600 mW/cm^2^. The μSBS of half of the specimens within each group (*n* = 10) was measured after 24 h of storage in distilled water at 37°C using a universal testing machine (Instron, Z020. Zwick Roell, Germany) at a crosshead speed of 1 mm/min and with a direction parallel to the bonded interface. The other half of the specimens were tested after 6 months of storage in distilled water at 37°C. The distilled water was changed weekly for 6 months. The diagram of the experimental design used in this study is summarized in Figure 1.

The failure mode analysis was performed under a stereomicroscope at ×40 magnification (Carl Zeiss Inc., Oberkochen, Germany) and classified as follows: (A) adhesive failure at the adhesive‐composite interface (adhesive remaining on dentin) or at the adhesive‐dentin interface (intact dentin surface and no composite remnants) or; (B) cohesive failure in the composite/dentin; and (C) mixed failure including areas of cohesive failure within dentin, and/or composite combined with areas of adhesive failure (Lezaja et al., 2016) .

The normality of data distribution was evaluated by Kolmogorov–Smirnov test. Afterward, data analysis was performed using ANOVA, Tukey's post‐hoc test, and independent *t*‐tests. The Statistical Package for the Social Sciences (SPSS version 17) software (SPSS Inc., Chicago, USA) was used for data analysis and *p* values less than 0.05 were considered significant.

## 3 RESULTS

The mean μSBS values and standard deviations for all groups are depicted in Table 1. Kolmogorov–Smirnov test showed that the data distribution in all experimental groups was normal.

**TABLE 1:** Mean microshear bond strength (μSBS) ± SD of the Adper single bond adhesive system to CAD obtained in each experimental condition

Experimental condition	24 h	6 m	*p* value
Control (no pretreatment)	21.86 ± 1.6^Aa^	17.78 ± 1.6^Ba^	0.001
CPP‐ACP	22.34 ± 1.5^Aa^	15.40 ± 3.7^Bba^	0.012
PA	24.44 ± 1.6^Ab^	22.93 ± 3.9^Ac^	0.277
PA + CPP‐ACP	22.90 ± 1.8^Aab^	20.17 ± 2.8^Bca^	0.015
*p* value	0.005	0.001	

*Note:* Mean values with different uppercase superscript letters indicate statistically significant differences within each row (independent *t*‐test, *p* < 0.05). Mean values with different lowercase superscript letters indicate statistically significant differences within each column (Tukey's post‐hoc test, *p* < 0.05).

Abbreviations: CAD, Caries affected dentin; CPP‐ACP paste, casein phosphopeptide‐amorphous calcium phosphate paste; PA, proanthocyanidin.

A two‐way ANOVA showed that all 2‐way interaction effects were statistically significant (all *p* values <0.05). Subgroup analysis was performed to compare μSBSs among different groups in each time (24 h and 6 months) using one way ANOVA followed by Tukey's post‐hoc test. Moreover, independent *t*‐tests were performed to compare the differences between the μSBSs of the groups after 24 h and 6 months of water storage.

One‐way ANOVA revealed significant differences regarding the μSBS of the groups after 24 h (*p* < 0.05). The μSBS of the PA‐pretreated group was significantly higher than those of the control and CPP‐ACP groups (*p* values <0.05). There was no significant difference between the μSBS of the control group and CPP‐ACP or PA + CPP‐ACP groups. No significant difference was found in the μSBS of the group pretreated with PA + CPP‐ACP and those of other groups (*p* values >0.05).

One‐way ANOVA also revealed significant differences among the groups after 6 months (*p* < 0.05). The PA group showed a significantly higher μSBS compared with the control and CPP‐ACP groups (*p* < 0.05). However, no significant differences were found regarding the μSBS of the control group and the groups pretreated with CPP‐ACP and PA + CPP‐ACP (*p* values >0.05). Also, the μSBS of the PA + CPP‐ACP group was significantly higher than that of the CPP‐ACP group (*p* < 0.05). No significant difference was found between the PA‐pretreated group and the PA + CPP‐ACP group (*p* values >0.05).

The μSBS of the 6‐month specimens was significantly lower than those of the 24‐hour specimens for all the groups (*p* < 0.05) except for those pretreated with PA which did not exhibit a significant difference between 24 h and 6 months of water storage (*p* > 0.05).

According to the failure mode analysis, there was no significant difference among the experimental groups and the most common type of failure was mixed failure (Table 2).

**TABLE 2:** The number of specimens according to the fracture mode of each group

Experimental condition	Failure mode					
	24 h			6 months		
	A	C	M	A	C	M
Control (no pretreatment)	2	0	8	2	2	6
CPP‐ACP	1	1	8	2	1	7
PA	1	0	9	1	2	7
PA + CPP‐ACP	2	0	8	1	1	8

*Note:* Modes of failure: A, adhesive failure; C, cohesive failure; M, Mixed.

## 4 DISCUSSION

This study was conducted to evaluate the effect of dentin pretreatments with CPP‐ACP and PA on the μSBS durability of an etch‐and‐rinse adhesive to CAD. It was found that the μSBS decreased in all experimental groups (except for the PA‐pretreated group) after 6 months of water storage.

The loss of the integrity of the dentin collagen matrix of CAD results in a lower adhesive bond strength to CAD compared to that of normal dentin (Doozandeh et al., 2015). The intertubular dentin in CAD is partially demineralized. Because of the remineralizing effect of the CPP‐ACP (Poggio et al., 2013), it was used to pretreat CAD in the present study. Based on the results of the present study, a 3‐min pretreatment of CAD with CPP‐ACP did not affect the μSBS of the adhesive to CAD after 24 h or 6 months which is in line with a previous study (Doozandeh et al., 2015). More calcium precipitates already exist within the dentinal tubules of the CAD compared to normal dentin (Shimizu et al., 1981). In spite of the positive effect of the CPP‐ACP application on the remineralization of the partially demineralized intertubular dentin in CAD, it might have a negative effect on the already existing calcium precipitates within the dentinal tubules of CAD and lead to their further occlusion with calcium precipitates. This dual effect of the CPP‐ACP may interfere with adhesive resin penetration into dentinal tubules and decrease the permeability of dentinal tubules which finally results in neutralizing the positive effect of CPP‐ACP on the remineralization of the intertubular dentin in CAD. Further studies are needed to investigate the exact role of CPP‐ACP on the mineral content of the CAD.

Another agent used as dentin pretreatment on CAD in the current study was PA. It has been suggested that PA, in conjunction with fluoride, can be used to prevent caries (Pavan et al., 2011). Although PA incorporation directly into dental adhesives was able to increase the substantivity of PA in the hybrid layer and enhanced its collagen cross‐linking effect, it reduced the optimum quality of the hybrid layer. This finding was attributed to the radical scavenging ability of PA resulting in a low degree of double bond conversion (D. Epasinghe, Yiu, et al., 2012). Moreover, the inclusion of PA in dental adhesives could interfere with the light‐curing of the adhesive resins (Y. Liu & Wang, 2013). Therefore, PA was used for CAD pretreatment before adhesive application in the current study. The groups pretreated with PA demonstrated higher μSBS to CAD after 24 h and 6 months of water storage compared to the control groups and the groups pretreated with CPP‐ACP in this study. This is in accordance with the findings of a previous study which demonstrated that the application of 6.5% PA to dentin significantly improved the immediate bond strength of composite to dentin (Macedo et al., 2009). This result might be justified by the collagen crosslinking and stiffening following CAD pretreatment with PA. Furthermore, it was demonstrated that the dense collagen network formed by the use of cross‐linkers such as PA reduced water absorption of dentin and improved the mechanical properties of demineralized dentin such as modulus of elasticity and hardness (C. S. Castellan Pereira, et al., 2010). Also, PA can play an important role in collagen biosynthesis (Y. Liu, Dusevich, & Wang, 2014). Therefore, PA can have positive effects on the inner remineralizable portion of the CAD which has decreased collagen cross‐links without denatured proteins. All of these different interactions might increase the remineralization and decrease the demineralization of the collagen matrix of the CAD.

In the present study, the combined use of PA and CPP‐ACP could not promote the μSBS to CAD when compared to the application of PA alone and a synergistic effect of PA and CPP‐ACP could not be observed. This may be due to the probable interaction between PA and CPP‐ACP. Also, the PA effect might be minimized by the precipitation of minerals resulting from CPP‐ACP on the surface of the CAD which could interfere with the remineralization of deeper parts of the CAD. Possibly, a different combination of application time and concentration of PA and CPP‐ACP can reduce mineral precipitation on the surface of the CAD, resulting in the improved synergistic effect of PA and CPP‐ACP on μSBS to CAD. However, this assumption needs further investigations.

Another finding of the current study was that the μSBS to CAD decreased after 6 months of water storage except for the PA group. This decrease might result from water sorption by the polymer which possibly causes the hydrolysis of the resin components and their subsequent degradation and leaching from the hybrid layer. Also, host‐derived enzymes exhibiting collagenolytic activity such as MMPs and exogenous proteinases can degrade collagen fibrils (Pashley et al., 2004). In the present study, the application of PA helped to maintain the bond strength between CAD and the adhesive resin after 6 months. The effect of PA on inhibiting the proteolytic activity on the demineralized CAD matrix and thus protecting the adhesive/dentin interface against enzymatic degradation can be an explanation for this finding (R.‐R. Liu, Fang, et al., 2014). PA can inactivate proteolytic enzymes in the dentin matrix by covalent bonds that are stable over time unlike the reversible electrostatic binding of chlorhexidine (C. S. Castellan, Pereira, et al., 2010). The stability of proanthocyanidin‐collagen interaction results from the formation of bridge‐type hydrogen bonds between the phenyl hydroxyl groups of PA and the side chains of carboxyl, hydroxyl, amino, and amide groups of the collagen molecules (Han et al., 2003). Also, the interaction between PA and collagen is based on the hydrophobic effect between the phenyl rings of PA and the pyrrolidine rings of proline. No pH‐sensitive group is involved in this interaction which makes it different from common chemical cross‐linkers such as glutaraldehyde (Y. Liu, Dusevich, & Wang, 2014). Therefore, the interactions between PA and collagen should not be compromised by an acidic environment (M. Firouzmandi, Vasei, et al., 2020; Y. Liu, Dusevich, & Wang, 2014). A previous study also showed that application of PA prior to the acid challenge resulted in the improvement of the mechanical properties of CAD and stabilization on collagen matrix (M. Firouzmandi, Vasei, et al., 2020).

According to the results of the present study, CAD pretreatment with PA can be recommended as an effective chairside procedure to overcome the disadvantage of reduced bond strength durability of composite resin to CAD. The results of the present study should be confirmed by analyses of the interface such as nanoleakage. Also, the possible relationship and interaction between PA and CPP‐ACP when used for CAD pretreatment with different adhesive bonding systems such as the self‐etch adhesives should be investigated in future studies. More studies with increasing storage time and/or adding collagenolytic enzymes could better reflect the efficacy of PA and CPP‐ACP in improving μSBS to CAD in clinical situations.

## 5 CONCLUSIONS

Due to the positive effects of PA pretreatment on the bond strength between the adhesive and CAD after 24 h and 6 months of water storage, PA can be recommended to enhance the longevity of composite resin restorations to CAD. The same results were not obtained for CPP‐ACP or simultaneous application of PA and CPP‐ACP.

## CONFLICT OF INTEREST

The authors declare no conflicts of interest.

## Data Availability

The data that support the findings of this study are available from the corresponding author upon reasonable request.
